# Hepatotoxic Seafood Poisoning (HSP) Due to Microcystins: A Threat from the Ocean?

**DOI:** 10.3390/md11082751

**Published:** 2013-08-05

**Authors:** Katerina Vareli, Walter Jaeger, Anastasia Touka, Stathis Frillingos, Evangelos Briasoulis, Ioannis Sainis

**Affiliations:** 1 Department of Biological Applications and Technologies, University of Ioannina, 45110 Ioannina, Greece; E-Mail: kvareli@cc.uoi.gr; 2 Interscience Molecular Oncology Laboratory, Human Cancer Biobank Center, University of Ioannina, 45110 Ioannina, Greece; E-Mails: atouka@cc.uoi.gr (A.T.); ebriasou@otenet.gr (E.B.); 3 Department of Clinical Pharmacy and Diagnostics, University of Vienna, A-1090 Vienna, Austria; E-Mail: walter.jaeger@univie.ac.at; 4 Laboratory of Biological Chemistry, School of Medicine, University of Ioannina, 45110 Ioannina, Greece; E-Mail: efriligo@cc.uoi.gr

**Keywords:** microcystin, Hepatotoxic Seafood Poisoning (HSP), cyanobacteria, *Synechococcus* sp., *Synechocystis* sp., marine environments, microcystin synthetase genes (*mcyS*)

## Abstract

Cyanobacterial blooms are a major and growing problem for freshwater ecosystems worldwide that increasingly concerns public health, with an average of 60% of blooms known to be toxic. The most studied cyanobacterial toxins belong to a family of cyclic heptapeptide hepatotoxins, called microcystins. The microcystins are stable hydrophilic cyclic heptapeptides with a potential to cause cell damage following cellular uptake via organic anion-transporting proteins (OATP). Their intracellular biologic effects presumably involve inhibition of catalytic subunits of protein phosphatases (PP1 and PP2A) and glutathione depletion. The microcystins produced by cyanobacteria pose a serious problem to human health, if they contaminate drinking water or food. These toxins are collectively responsible for human fatalities, as well as continued and widespread poisoning of wild and domestic animals. Although intoxications of aquatic organisms by microcystins have been widely documented for freshwater ecosystems, such poisonings in marine environments have only occasionally been reported. Moreover, these poisonings have been attributed to freshwater cyanobacterial species invading seas of lower salinity (e.g., the Baltic) or to the discharge of freshwater microcystins into the ocean. However, recent data suggest that microcystins are also being produced in the oceans by a number of cosmopolitan marine species, so that Hepatotoxic Seafood Poisoning (HSP) is increasingly recognized as a major health risk that follows consumption of contaminated seafood.

## 1. Introduction

Cyanobacterial toxins (cyanotoxins) belong to diverse chemical classes and can cause cell-specific toxic events, such as neurotoxicity by anatoxin-a, anatoxin-a(.) and saxitoxins, hepatotoxicity by microcystins, nodularins and cylindrospermopsin and dermatitis by lyngbyatoxin-a [1,2,3,4].

Following the drinking of contaminated water, livestock poisoning and adverse human health effects have been reported. The World Health Organization (WHO) has published both a guideline value for the most prevalent cyanotoxin in drinking water and a procedural guideline for recreational waters [5].

Among cyanotoxins, the cyclic hepatotoxins, nodularins (Nods) and microcystins (MCs), are the most common natural toxins [3,6]. Nodularins are produced only by strains of the genus, *Nodularia,* and thus, any reported poisoning due to Nods can be confidently attributed to this genus [7,8]. This is not the case for MCs, by far the most prevalent of the cyanobacterial toxins, which are produced by strains of distantly related genera, such as *Microcystis*, *Anabaena*, *Planktothrix* and, more rarely, *Anabaenopsis*, *Hapalosiphon* and *Nostoc* [6,9,10].

Microcystins are synthesized non-ribosomally by the thiotemplate functions of large multifunctional enzyme complexes containing both non-ribosomal peptide synthetase (PS) and polyketide synthase (PKS) [11]. The microcystin biosynthesis gene cluster (*mcyS*) has been sequenced and partially characterized in several cyanobacterial freshwater species [12,13,14]. In *Microcystis aeruginosa*, the microcystin biosynthesis gene cluster (*mcyS*) spans 55 kb, comprises 10 genes arranged in two divergently transcribed operons (*mcyA–C* and *mcyD–J*) and includes genes encoding peptide synthetases (*mcyA*, *mcyB* and *mcyC*), polyketide synthases (*mcyD*), hybrid PS–PKS enzymes (*mcyE*, *mcyG*) and enzymes putatively involved in the tailoring (*mcyJ*, *mcyF* and *mcyI*) and transporting (*mcyH*) of the toxin [12]. Comparison of *mcyS* gene clusters from different genera revealed differences both in gene arrangements and nucleotide sequences. For instance, comparison of *mcyE* gene from *Anabaena* strain 90 with *mcyE* genes sequenced from other freshwater cyanobacteria revealed 75% identity to *Microcystis aeruginosa* and 78% identity to *Planktothrix agardhii* [14]. The homologies are even lower for *mcyA* gene, 69% identity to *Microcystis aeruginosa mcyA* gene and 67% identity to *Planktothrix agardhii mcyA* gene (see Table 1). 

**Table 1:** Comparison of microcystin synthetase genes (G) and polypeptides (P) from different cyanobacteria species or strains with those sequenced from *Anabaena* strain 90. Percentages in parentheses referred to query coverage. References for the sequences: *Anabaena* sp. strain 90 [14]; *Microcystis aeruginosa* NIES-843 [15]; *Microcystis aeruginosa* UV027, Raps *et al*., unpublished work (GenBank accession number: AF458094) and Botes, E., unpublished work (GenBank accession number: AY034602); *Microcystis aeruginosa* PCC7806 [16]; *Planktothrix agardhii* [13]; *Planktothrix rubescens* NIVA-KYA 98 [17].

	*Microcystis aeruginosa* NIES-843		*Microcystis aeruginosa* UV027		*Microcystis aeruginosa* PCC7806		*Planktothrix agardhii*		*Planktothrix rubescens* NIVA-KYA 98	
	G	P	G	P	G	P	G	P	G	P
***mcyA***	71% (94%)	68% (99%)	71% (94%)	68% (99%)	71% (94%)	68% (99%)	67% (94%)	66% (99%)	67% (82%)	66% (84%)
***mcyB***	74% (99%)	72% (100%)	74% (99%)	72% (100%)	75% (99%)	69% (100%)	72% (99%)	70% (100%)	72% (99%)	70% (100%)
***mcyC***	76% (99%)	74% (99%)	75% (99%)	74% (98%)	75% (99%)	74% (99%)	81% (99%)	80% (99%)	81% (99%)	80% (99%)
***mcyD***	73% (99%)	69% (99%)			73% (99%)	69% (99%)	77% (99%)	73% (99%)	77% (99%)	73% (99%)
***mcyE***	76% (98%)	75% (100%)			75% (98%)	75% (100%)	79% (99%)	77% (100%)	79% (99%)	77% (100%)
***mcyF***	74% (90%)	69% (91%)			74% (90%)	70% (91%)				
***mcyG***	76% (97%)	71% (99%)			75% (97%)	71% (99%)	79% (97%)	75% (98%)	79% (97%)	75% (98%)
***mcyH***	76% (94%)	72% (96%)			76% (93%)	73% (95%)	79% (94%)	75% (99%)	79% (94%)	75% (99%)
***mcyI***	75% (95%)	74% (97%)			75% (94%)	72% (97%)				
***mcyJ***	81% (98%)	83% (89%)			81% (98%)	82% (89%)	81% (99%)	83% (88%)	81% (99%)	83% (88%)

Based on these sequence differences, Rantala and collaborators developed genus specific primer sets in order to identify putative toxic cyanobacterial genera in mixed freshwater cyanobacterial populations [18]. 

Microcystin production in toxic cyanobacteria is thought to be influenced by a number of different physical and environmental parameters, such as nitrogen, phosphorus, trace metals, temperature, light and pH [19,20,21,22]. Nevertheless, although the above mentioned environmental factors are associated with toxin production, no models exist that can predict toxin concentrations in natural ecosystems [10]. 

At the molecular level, high light intensities and red light were found to increase transcription of the *mcyS* gene cluster, while blue light led to reduced transcript levels [23]. Recently, it was found that NtcA, a transcription factor that has been characterized for a variety of cyanobacterial species, binds to the *mcyS* promoter from *M. aeruginosa* PCC 7806 [24]. While NtcA is produced at a basal level in the presence of ammonium and the level is elevated under nitrogen stress conditions [25,26,27,28], it has been suggested that the regulation of microcystin synthetase gene transcription is responsive to nitrogen.

The MCs comprise a series of more than 90 structural variants of a cyclic heptapeptide with the general structure, *cyclo*-(d)-Ala-X-(d)-*erythro*-β-methyl-*iso*-Asp-Y-Adda-(d)-*iso*-Glu-.-methyldehydro-Ala. The letters X and Y represent positions that are occupied by variable l-amino acids. [10,29,30,31]. The most toxic and most studied MC variant is MC-LR [a variant in which the two variable amino acids are leukine (L) and arginine (R)], a known hepatotoxin that preferentially affects liver cells. The hydrophilic structure of most MC variants hinders their penetration through the plasma cell membrane and, therefore, requires uptake via active transport. Active transmembrane transport of MCs is mediated by organic anion transporting polypeptides (human OATP/rodent Oatp) [32]. Approximately 80 OATPs/Oatps have been found in different species [33]. Based on different affinities for MCs, only a few OATPs/Oatps are able to transport MCs [34]. Relatively recent data showed that at least the ubiquitously expressed OATP1A2 and the liver-specific OATP1B1 and OATP1B3 are active transporters for various MC variants, especially for MC-LR [32]. Therefore, the liver is their main target, and these toxins have been traditionally considered as hepatotoxins [32]. It must be noticed that OATPs are not only expressed in the liver, but also in the gastrointestinal tract, kidney and brain, and there is evidence that MC-LR can be transported across the human blood-brain barrier (BBB) [32,35,36]. Indeed, several OATPs/Oatps have been already identified in the BBB, the human gliomas and glioma cells, indicating that MCs are able to enter the brain and exert neurotoxic effects [37,38]. Upon entrance to the OATPs-expressing cells, microcystins bind directly to PP1 and PP2A catalytic centers and blocking the access completely of the substrate, resulting in inhibition of the enzyme activity [39]. Since PP2A has been shown to regulate the activity of at least 50 protein kinases, such as protein kinase C (PKC), Akt, extracellular signal-regulate kinase (ERK), mitogen-activated protein kinase (MAPK), IκB kinase, p38, and caspase-3, it is reasonable that the effect of an inhibitor of PP2A activity is detrimental for the cell [40,41,42,43]. Recently, it has been shown that under conditions of p53 inactivation, chronic exposure to low doses of microcystins may lead to cell proliferation through activation of Akt signaling [44]. In hepatocytes, the most apparent effect is the morphological transformation of microtubules [45,46]. Livers of intraperitoneally and orally treated rodents become swollen and hemorrhagic. Extensive dissociation and disruption of liver epithelium have been reported. [47,48,49]. Oxidative stress has been shown to play a role in microcystin-induced cytotoxicity [50]. It has been observed that MCs exert genotoxic effects, such as DNA fragmentation, chromosomal aberrations, micronuclei formation, loss of heterozygosity and even base substitution mutations [51,52,53,54,55] and that sublethal doses of MC-LR can result in hepatocellular apoptosis [56]. 

In chronic low dose exposure, MCs are thought to exert tumor-promoting effects also through inhibition of the PP1 and PP2A, which are known to function as tumor suppressors [57]. 

Detoxification of MCs in both mammals and aquatic organisms are facilitated through glutathione (GSH) and cysteine (Cys) conjugation pathways [58,59,60]. For example, the GSH and Cys metabolites have been identified in mouse and rat liver treated with MCs. Interestingly, injection of both GSH and Cys conjugates to mice showed reduced toxicity compared to native MCs, but their toxicity still remains [58]. Very recently, it was demonstrated that cyanobacteria-eating bighead carps metabolizes MC-LR and MC-RR [a variant in which both variable amino acids are arginine (R)] mainly to Cys conjugates with much higher concentrations in kidneys than in liver, intestine and muscle, suggesting a possible organotropism to kidneys in fish [61,62].

Although a considerable amount of information is available on the effects of MCs on human and vertebrate hepatocytes [63], the ecological function of microcystins remains unresolved. 

Initially, it was believed that MCs had evolved in response to grazing pressure by zooplankton, but there is recent evidence that their production by cyanobacteria may actually predate the metazoan lineage [64]. This suggests that the toxins would have evolved to serve other functions, such as response to infochemicals released by herbivorous zooplankton, scavenging of trace metals or signaling and gene regulation [65,66,67,68,69]. Recently, a role of MC as a protein-modulating metabolite and protectant against oxidative stress has been suggested [70]. If these are the primary and original functions of this secondary metabolite, then it would explain why probably all ancestral cyanobacteria produced these heptapeptides [64]. 

The sporadic distribution of *mcyS* genes in modern cyanobacteria suggests not only that the ability to produce the toxin has been lost repeatedly during evolution, but also that cyanobacteria species or strains not previously suspected of producing microcystins may retain the genes necessary for their synthesis. This would be important for strains of genera, like *Spirulina*, *Arthrospira*, and *Aphanizomenon*, that are commonly used in health food supplements [71]. 

In addition, according to a constructed phylogenetic tree [64], *Synechococcus* sp. and *Synechocystis* sp. strains are also members of the cyanobacteria lineage, which originally possessed *mcyS* genes. This is of particular importance, provided that *Synechococcus* and *Synechocystis* genera are among the most common cyanobacteria, not only in freshwaters, but also in the world ocean. Moreover, it is noteworthy that the cosmopolitan open ocean cyanobacterium, *Synechococcus* sp., has been reported to form persistent blooms in coastal waters worldwide [72].

## 2. Microcystins in the World Ocean

**Figure 1:**
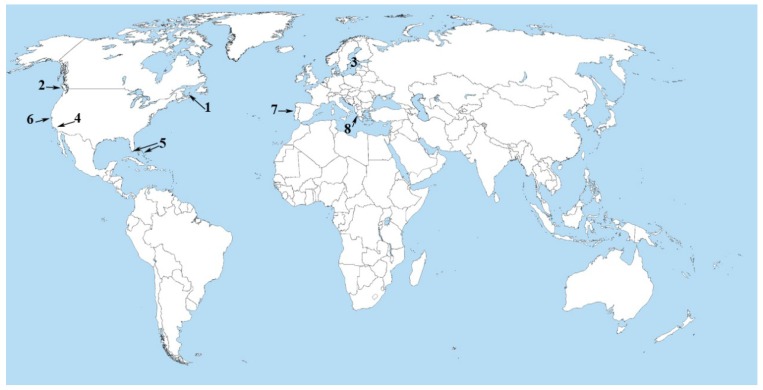
Major appearances of microcystins in the world ocean. (**1**) Prince Edward Island (Eastern Canadian coast), (**2**) Gillam Island (Northwestern Pacific coast), (**3**) Baltic Sea, (**4**) Salton Sea, (**5**) Florida Keys and Bahamas, (**6**) Monterey Bay, (**7**) Portuguese coast, (**8**) Amvrakikos Gulf (Mediterranean Sea). For references and details, see the text.

The presence of toxins of the microcystin family in oceanic and coastal waters was identified initially in mussels collected from the Northeastern Pacific, European and eastern Canadian coasts (Figure 1) [73].

More specifically, MC-LR was identified in mussels from Gillam Island (Northeastern Pacific) (Figure 1), Nod in European (Dutch) mussels and both of them in mussels harvested from Prince Edward Island (eastern Canadian coast) (Figure 1) [73]. Moreover, the proximity of Gillam Island to a net pen aquaculture site (Winter Harbor, British Columbia), where commercially reared Atlantic salmon were found to be suffering from a liver disease (net pen liver disease) characterized by severe necrosis and hepatic megalocytosis, led to the notion that microcystins could be implicated in the etiology of the disease [74]. Interestingly, liquid chromatography analysis of liver tissue extracts taken from Atlantic salmon afflicted with net pen liver disease showed the presence of an inhibitor of PP that was chromatographically indistinguishable from MC-LR [74]. Intraperitoneal injection of MC-LR into healthy Atlantic salmon re-created the pathologic changes of net pen liver disease, including diffuse necrosis and hepatic megalocytosis [74]. Unfortunately, the organisms responsible for production of MCs found in the above mentioned studies remain unidentified, but, as suggested by the authors, are likely to be marine prokaryotes.

Nodularin contamination of mussels, prawns and fish has been repeatedly reported [6,7]. Blooms of the toxic marine/brackish species, *Nodularia spumigena*, are a common issue during the summer months in the Baltic Sea and in estuarine lakes in Australia and New Zealand [6,7,75,76]. While Nod is produced only by the cyanobacterium, *Nodularia*, it is reasonable to assume that Nod identified in Dutch and eastern Canadian mussels [73] is produced by *Nodularia* sp. 

In contrast, it is difficult to speculate on the origins of MCs in contaminated mussels harvested from the Northeastern Pacific and Canadian coasts.

Recently, it was found that in addition to Nod, MC-LR levels are high in the Baltic Sea (Figure 1) [77], and the most probable candidate organism for microcystin-LR production is thought to be *Anabaena flos-aquae*, rather than *Anabaena lemmermannii* [78]. It should be noted that both *Anabaena flos-aquae* and *Anabaena lemmermannii* are freshwater species. The Baltic Sea (Figure 1) is one of the world’s largest brackish water environments. Negligible tidal influences and a combination of nutrient rich freshwater inflows and limited water exchange with the North Sea generate freshwater-saltwater gradients [79,80], which allow freshwater cyanobacteria to establish cyanobacteria blooms. Accumulating evidence for the presence of MCs in the Baltic Sea led to the notion that MCs in marine environments are produced by freshwater invading species. 

There is also another possible explanation for the presence of MCs in marine environments: ocean discharge of MCs produced in upstream freshwater lakes and reservoirs. Along the Pacific coast of the United States, large-scale *Microcystis* blooms with toxin production occur each year in lakes and rivers throughout Washington [81], Oregon [71] and California [82,83]. In California, *Microcystis*-contaminated runoff has been documented at the marine interfaces of the Klamath River [84] and San Francisco Bay [85], but the health hazards associated with MCs have received public attention only recently, after numerous documented marine mammal deaths linked to MC intoxication [86].

Deaths of 21 sea otters from Southern California, a federally listed threatened species, were linked to MC intoxication and connected to the ocean discharge of freshwater MCs from three nutrient-impaired rivers flowing into the Monterey Bay National Marine Sanctuary (Figure 1) [86]. Moreover, MC concentrations up to 2900 ppm (one of the highest microcystin concentrations ever reported from an environmental sample) were detected in a freshwater lake (Lake Pinto) and downstream tributaries to within 1 km of the ocean [86]. The MC producing cyanobacterium in Pinto Lake was found to be *Microcystis* sp. Microcystins soluble in water, and toxic *Microcystis* sp. bloom material from Lake Pinto continuously feeds Monterey Bay by the rivers. A laboratory experiment revealed that *Microcystis* sp. cells are lysed within 48 hrs after exposure to seawater, subsequently releasing microcystins [86]. Microcystin-LR was detectable in seawater for the duration of the study (21 days), and MC concentrations in refrigerated seawater had declined only 44%–71% from the mean one hour post-exposure levels after three weeks. Significant bioaccumulation and slow depuration of freshwater MCs by marine oysters, clams, snails and mussels, with gastrointestinal tissue concentrations up to 107-times greater than adjacent seawater were detected [86]. High MC concentrations were detected in marine invertebrates even two weeks post-exposure. Under the microscope, livers of microcystin-positive sea otters exhibited hepatocellular vacuolation, apoptosis, necrosis and hemorrhage consistent with previous descriptions of microcystin intoxication in humans and animals [87,88]. Based on these results, the authors concluded that MCs cause mortality of threatened southern sea otters, through the consumption of contaminated marine invertebrate prey [86]. 

If we reconsider the first published (1993) work on the presence of MCs in Northeastern Pacific mussels [73], it would be reasonable to assume that this presence could have had a freshwater origin and that ocean discharge of contaminated freshwater would have been the most probable route of exposure. The discussion about the origins of MCs in marine environments could have ended here, if a number of recent publications had not appeared to support the notion of a health threat hazard from the ocean itself, distinct from the well documented threat from the inland waters.

## 3. The Black Band Disease of Corals

Black band disease (BBD) is a migrating, cyanobacterial dominated, sulfide-rich microbial mat that moves across coral colonies lysing coral tissue [89]. Although it is known that BBD sulfate-reducing bacteria contribute to BBD pathogenicity by production of sulfide, the toxin MC was detected in 22 field samples of BBD collected from five coral species on nine reefs of the wider Caribbean (Florida Keys and Bahamas) (Figure 1) [89]. Two cyanobacterial cultures isolated from BBD, *Geitlerinema* sp. and *Leptolyngbya* sp., were found to be microcystin producers. The *mcyA* gene from the microcystin synthesis complex was detected in two field samples and from both BBD cyanobacterial cultures. Interestingly, the *Geitlerinema*’s *mcyA* gene fragment (EF432064) was found to be 100% homologous only in a small part of the total sequence (3% query coverage) of the *M. aeruginosa mcyA* gene [89]. The same was found for *Leptolyngbya*’s *mcyA* gene fragment (EF432065, 100% homologous, 8% query coverage of *Planktothrix* NIVA-CYA34 *mcyA* gene). While the PCR amplified products from *Geitlerinema* and *Leptolyngbya* cultures were 608 and 285 bp-long, respectively, a 100% homology along with 3%–8% query coverage to known *mcyA* genes means that homology was restricted only to the primers that have been used. Given that *mcyS* genes are well preserved during evolution [64,90] and the primers that have been used in this study are well characterized [91,92], the isolation of putative *mcyA* gene fragments from MC producing axenic cyanobacterial cultures, with low homology to known *mcyA* genes, is rather peculiar. Does it mean that there are differences between “freshwater” and “marine” *mcyA* genes? 

In a more recent study [93], two cultured laboratory strains of BBD *Oscillatoria* sp., isolated from reefs of the wider Caribbean, were analyzed for MC production using Ultra-Performance Liquid Chromatography-Tandem Quad Mass Spectrometry (UPLC-MS/MS). The cyanotoxin MC-LR was found to be produced by these strains. However, no effort was made in this study to amplify a *mcyS* gene fragment from *Oscillatoria* sp. cultures.

## 4. Back to the Land—The Salton Sea

The Salton Sea is the largest inland body of water in California (Figure 1), with salinities varying from freshwater/brackish water at the major river outlets to hypersaline conditions in the sea proper [94]. Over the past 15 years, wintering populations of eared grebe (*Podiceps nigricollis*) at the Salton Sea have experienced over 200,000 mortalities. The cause of these large die-offs remains unknown [94]. An investigation to determine the role of cyanotoxins in grebe mortalities and morbidities on the Salton Sea was started in November 1999 and ended in April 2001. The results were published in 2006 [94]. Microcystins were detected with ELISA in the majority of liver and intestine samples from grebes collected, due to morbidity or mortality from the Salton Sea. Isolation, culture and ELISA testing for microcystin of 50 strain isolates showed that MCs were produced by all strains—albeit at low levels (<1 mg g^−1^ dry weight) [94]. The genera producing measurable levels of MCs included mainly the planktonic species, *Synechococcus* sp., and the benthic filamentous species, *Oscillatoria* sp. The most notable result of the study was that a microcystin-producing *Synechococcus* strain (SS-1) was found by 16S rRNA analyses to be closer to marine strains of *Synechococcus* than to freshwater strains of this genus. Moreover, *Synechococcus* isolates from the Salton Sea were shown to produce both MC-LR and MC-YR [a variant in which the two variable amino acids are tyrosine (Y) and arginine (R)], by LC/MS [94]. Marine *Synechococcus* accounted for 50% of the total cyanobacterial biomass in the world ocean, and that is why the authors stated that the documented production of MCs by a marine-like *Synechococcus* species indicates that the presence of the toxin in marine environments should be more common than previously thought.

On the other hand, in this study [94], no attempt was made in order to characterize potential *mcyS* genes from the cultivated marine-like microcystin producing *Synechococcus* SS-1 strain, and thus, we cannot speculate about the homologies between “marine” and “freshwater” *mcyS* genes.

## 5. Atlantic Ocean (Portuguese Coastal Waters)

Common marine cyanobacteria strains of *Oscillatoria*, *Synechocystis* and *Synechococcus* genera were isolated from Portuguese coastal waters (Figure 1), and their extracts were found to cause toxic effects on mice [95]. Hepatocellular necrosis, pycnotic nuclei, dilatation of the sinusoidal spaces, cytoplasmatic vacuolation with loss of nuclei and granulovacuolar degeneration with perinuclear clumping of cytoplasm were the signs of hepatic toxic effects detected after intraperitoneal injection of some crude cyanobacterial extracts in mice [95]. Taking into account that similar effects were reported earlier [96] for the intoxication with microcystins, the authors screened the cyanobacterial strains for microcystins by ELISA. Seven of them (four *Synechococcus* strains, one *Synechocystis* strain, one *Oscillatoria* strain and one *Cyanobacterium stanieri* strain) were found to produce MCs, albeit in small quantities [95]. Nevertheless, there was no clear relation between the levels of MCs measured by the ELISA and the histopathologies observed. The authors hypothesized that although the ELISA method is too specific for a number of MC variants, there is always the possibility of the presence of other variants with lower specificities to the ELISA kit that has been used [95]. Alternatively, while similar histopathological effects were reported earlier by Teneva and collaborators for mice injected with an extract of *Lyngbya aerogineo-coerulea* that did not produce microcystins and for hepatotoxicity caused by unknown cyanobacterial hepatotoxins on a study using a freshwater species of the genus, *Phormidium* [97,98]. they concluded that other compounds, rather than the well-known hepatotoxins, should be produced by the Portuguese costal cyanobacterial strains [95]. In any case, cyanobacteria of the referred genera, namely *Synechocystis* and *Synechococcus*, have rarely been studied with respect to toxicity and, consequently, to the potential threat to human and environmental health. The ability of the strains to produce toxins was evident, and the study conducted by Martins and collaborators was the first report about the toxic potential of these genera [95]. The importance of their study is reinforced by the fact that both *Synechococcus* and *Synechocystis* genera are ubiquitous in the world ocean. On the other hand, the authors did not try to isolate gene fragments corresponding to the *mcyS* gene cluster in those strains where MC concentrations were measurable. 

This was, however, done by the same group recently [99], while trying to answer the question: are known cyanotoxins involved in the toxicity of picoplanktonic and filamentous North Atlantic marine cyanobacteria? 

In this study, eight marine cyanobacteria strains of the genera, *Cyanobium* (3 strains), *Leptolyngbya* (2 strains), *Oscillatoria* (1 strain), *Phormidium* (1 strain) and *Synechococcus* (1 strain), were isolated from rocky beaches along the Atlantic Portuguese central coast, and the bioactivity of dichloromethane, methanol and aqueous extracts was assessed by the *Artemia salina* bioassay. Strains were toxic to the brine shrimp, *A. salina* nauplii, with aqueous extracts being more toxic than the organic ones [99].

Mass spectrometry analysis of the cyanobacterial extracts did not reveal the production of MCs or other known toxic peptides. Nevertheless, the authors analyzed the key genes involved in the production of known cyanotoxins, such as microcystins, nodularins and cylindrospermopsin [99]. To address this issue, they used one primer set for *mcyA* gene, one primer set for both microcystin and nodularin . gene and two primer sets for two different regions of the cylindrospermopsin synthetase gene cluster [99]. The primer set used in this study for *mcyA* gene amplification was the same as that used by Richardson and collaborators [89] in order to identify potential *mcyA* gene fragments from the MC producing BBD *Leptolyngbya* strain. While Richardson and coworkers [89] amplified a *mcyA* gene fragment with low similarity to known *mcyA* genes under low annealing conditions, Frasão and collaborators did not manage to amplify any *mcyA* gene fragment following the suggested annealing conditions [99]. In contrast, they managed to amplify fragments of *mcyE* genes by using primers specific for both MCs and Nods in two cases: in one *Leptolyngbya* strain and one *Oscillatoria* strain. Sequencing of the positive PCR products (GenBank accession numbers: HM124567 and HM124566) revealed 99% similarity to *Microcystis* sp. CYN06 *mcyE* gene, a similarity astonishingly high for distantly related genera [99]. 

In the case of the Portuguese strains [99], the identification of *mcyE* and not *mcyA* gene fragments along with the inability of the strains to produce MCs led the authors to hypothesize, based on the literature [64], that deletion events occurred during evolution, resulting in the loss of genes. Besides that, mutations may have occurred during cultivation of strains, as described earlier [100]. 

## 6. Mediterranean Sea (Amvrakikos Gulf, Greece)

In a recent study [101], MC concentrations were measured by ELISA, both in water and in the edible species of mussels, *Mytilus galloprovincialis*, collected from Amvrakikos Gulf, Greece (Figure 1), a Mediterranean marine ecosystem. Microcystin concentrations in *M. galloprovincialis* mussels exceeded the upper limit of the tolerable daily intake (TDI) of MC as determined by the World Health Organization [102]. The presence of MCs in the Gulf was undoubtedly identified by LC/MS analysis and the most prominent variants found to be MC-LR and MC-YR [101]. In order to identify potential MC producing cyanobacterial species in the Gulf, the authors used PCR-DGGE (Denaturing Gradient Gel Electrophoresis) profiling of the ITS sequences of the total cyanobacterial community. The cyanobacterial community was found to be dominated almost exclusively by the cosmopolitan genera, *Synechococcus*–*Synechocystis* [101]. In order to isolate putative *mcyS* gene fragments, the authors used a number of primer sets against any known freshwater *mcyA* and *mcyE* gene. Because it was known that PCR would fail to detect *mcyS* genes present in low abundance [89,103], they used as a template DNA extracted from a sample with a MC concentration high enough to reach the limit of detection, as set earlier by the same group [104,105]. Even though, the authors failed to amplify any PCR product. The inability to amplify PCR products in their environmental samples led to the notion that there must be differences between “freshwater” and “marine” *mcyS* genes [101]. Moreover, according to the authors, the lack of PCR products is strong evidence that MC presence in the Gulf is more likely to have a marine origin.

In an effort to isolate a putative *mcyS* gene fragment, an alternative procedure by using degenerated primers against PKS and PS of all freshwater cyanobacteria genera was followed. Indeed, a number of gene fragments corresponding to putative PKS or PS were isolated. Among them, there was one fragment with reasonable similarity to a known *mcyS* gene (*mcyG*) [101]. Given the overall prevalence of *Synechococcus* genotypes in Amvrakikos Gulf, phylogenetically clustered into five (subcluster 5.3, clade I, VI, WPC1 and WPC2) out of 18 previously identified clades [106,107], research efforts should be focused on worldwide isolates of these clades [101]. 

Interestingly, *Synechocystis*-like genotypes were found to be present only during periods of high MC contents in seston samples [101]. The similarity of ITS sequences among *Synechocystis*-like sequences from Amvrakikos Gulf and the closest relatives in GenBank were found to be low (*ca*. 85% to *Synechocystis* sp. PAK12, EF555570.1) [108]. Thus, uncharacterized uncultivated marine *Synechocystis* species should be considered also as putative MC producers.

## 7. Conclusions

Hepatotoxic Seafood Poisoning (HSP) risk upon consumption of MC contaminated seafood is increasingly documented. The presence of the cyanobacterial hepatotoxin, MC, in the marine environments is usually attributed to ocean discharge of toxin containing freshwaters or to freshwater toxin producing genera invading regions of lower salinity in the world ocean. In contrast, there is direct evidence for the existence of a marine-like MC-producing *Synechococcus* strain from a saline lake (Salton Sea, California-USA). Seawater cyanobacterial genera were also found to be microcystin producers, at least in one case, in the BBD disease of corals. Moreover, indirect evidence correlates the presence of the toxin in a Mediterranean marine ecosystem with the cosmopolitan cyanobacterial species, *Synechococcus* and *Synechocystis*. To date, no *mcyS* gene with reasonable homology to known freshwater counterparts had been isolated from a marine MC producing cyanobacterium, raising questions about the extent of sequence homologies between “freshwater” and “seawater” *mcyS* genes. Recent studies suggest that further investigation of *Synechococcus* and *Synechocystis* isolates as putative MC producers is of great importance, not only from an environmental health perspective, but also from an evolutionary one.
